# Upregulated FADD is associated with poor prognosis, immune exhaustion, tumor malignancy, and immunotherapy resistance in patients with lung adenocarcinoma

**DOI:** 10.3389/fonc.2023.1228889

**Published:** 2023-08-21

**Authors:** Miao He, Yingying He, Jian Xu, Youcai Zhang, Xiaoyu Cao, Li Wang, Feng Luo

**Affiliations:** ^1^ Lung Cancer Center, West China Hospital, Sichuan University, Chengdu, China; ^2^ Department of Oncology, Deyang People’s Hospital, Chengdu, China

**Keywords:** lung adenocarcinoma (LUAD), Fas-associated death domain protein (FADD), prognosis, immune microenvironment, Immunotherapy

## Abstract

**Background:**

FAS-associated death structural domain (FADD) proteins are important proteins that regulate apoptosis and are also involved in many nonapoptotic pathways in tumors. However, how dysregulated FADD affects the development of lung adenocarcinoma (LUAD) remains unknown.

**Method:**

Transcriptome profiles and corresponding clinical information of LUAD patients were convened from different databases, and the results were validated by qRT−PCR and cell counting kit-8 using LUAD cell lines. Potential associations between FADD and tumor malignancy, the immune microenvironment, genomic stability, and treatment sensitivity in LUAD patients were revealed by systematic bioinformatics analysis.

**Results:**

FADD was significantly overexpressed in LUAD, and patients with higher expression levels of FADD had a worse prognosis and more advanced tumor stage. Functional analysis revealed that elevated expression of FADD was associated with cell cycle dysregulation, angiogenesis, and metabolic disturbances. In addition, overexpression of FADD was associated with a higher infiltration of suppressive immune cells. From a single-cell perspective, cells with lower FADD expression are more active in immune-related pathways. FADD was associated with more genomic mutations, especially TP53. Patients with high FADD expression are more likely to benefit from conventional chemotherapy, while those with low FADD expression are more suitable for immunotherapy.

**Conclusions:**

Upregulated FADD is associated with worse prognosis, immune exhaustion, and tumor malignancy in LUAD patients. In addition, FADD can be an efficient indicator for assessing sensitivity to chemotherapy and immunotherapy. Therefore, FADD has the potential to serve as a new target for precision medicine and targeted therapy for LUAD.

## Introduction

Lung cancer is currently one of the most common malignancies worldwide and is the leading cause of death due to cancer (1). In particular, lung adenocarcinoma (LUAD) is the most common histopathological type of lung cancer, accounting for approximately more than half of all diagnosed lung cancer cases (2). In recent years, the incidence and mortality rates of LUAD have remained elevated, although advances in medical technology and clinical protocols have driven advances in diagnostic and therapeutic approaches (2). One of the biggest existing challenges is that the symptoms of early-stage LUAD are not obvious, resulting in many patients being diagnosed at an advanced stage and unfortunately missing the best opportunity for treatment. Fortunately, the development of molecularly targeted drugs and immunotherapies has led to more possibilities for the treatment of advanced LUAD (3). In addition, big data approaches and technologies of whole genome precision sequencing have provided breakthrough tools for the study of the pathogenesis and progression of LUAD, offering the possibility of individualized precision medicine (4). However, despite some advances, prevention, early diagnosis and treatment of LUAD still face great challenges due to its complexity and heterogeneity.

FADD (Fas-associated death domain protein) is an intracellular protein that plays a key role in the process of programmed cell death or apoptosis (5). FADD interacts with various proteins through its death domain (DD) and death effector domain (DED), leading to the activation of the caspase protein family and ultimately inducing apoptosis (6). In many cases, excessive activity or inhibition of FADD can have an effect on cell survival, especially in cases of cancer, immune diseases, and viral infections (7). For example, FADD expression may be increased in certain types of solid tumors (such as LUAD, glioma, and hepatocellular carcinoma), which may lead to enhanced cell death signaling and prevent the growth of cancer cells (8). Conversely, deletion or decreased expression of FADD may lead to cellular resistance to apoptotic signaling, which can lead to tumor development and growth (8). For example, FADD expression levels may be decreased in certain types of cancer, which may lead to resistance of tumor cells to apoptotic signaling (5). In these cases, restoring or increasing the expression of FADD may help restore sensitivity to apoptotic signals and stop further tumor development (5).

Immunotherapy is a revolutionary approach to cancer treatment designed to attack cancer cells by enhancing or altering the patient’s own immune system (9). FADD may influence the immune system’s response to cancer by regulating T-cell activity and viability (10). For example, the absence or loss of function of FADD may lead to reduced T-cell activity, which in turn may affect the effectiveness of immunotherapy (10, 11). In other studies, FADD activity has also been found to be associated with the immune escape mechanism of cancer cells. For example, certain types of cancer may resist apoptosis mediated by cytotoxic T cells or natural killer cells by reducing FADD activity (12). In recent years, studies targeting FADD have provided many important insights into its function and regulatory mechanisms, as well as the possibility of designing potential therapeutic strategies for cancer and other diseases (8). However, further studies are still needed to fully understand the specific role of FADD in cellular physiological and pathological processes.

Based on multiple LUAD datasets and a pancancer database, this study systematically explored the potential of FADD as an oncogene and prognostic marker. The correlation of FADD expression with the prognosis of LUAD patients, potential pathogenic mechanisms, dysregulated biological pathways, immune exhaustion, genomic instability, and therapeutic sensitivity was further evaluated. Our study aims to provide potential evidence to support novel precision medicine and targeted therapies for LUAD.

## Material and methods

### Data access and processing

We collected compliant LUAD sequencing datasets from two different databases. First, the count matrix of the TCGA-LUAD dataset, the maf format files of genomic mutant sites, and the corresponding clinical information were collected from the UCSC-Xena database (https://xena.ucsc.edu/). We included only patients without missing clinical information, containing a total of 492 LUAD patients, and used the count matrix as a development cohort after normalizing it to a transcripts per kilobase million (TPM) matrix. In addition, we collected the pancancer dataset from UCSC Xena. Three compliant LUAD datasets were retrieved from the GEO database (http://www.ncbi.nlm.nih.gov/geo/) and convened: GSE30219 (GPL570 Platform), GSE42127 (GPL6884 Platform), and GSE72094 (GPL15048 Platform). After including patients diagnosed with LUAD among them, the “sva” package was used to integrate the three datasets to remove batch effects between platforms (13), resulting in a meta-GEO dataset of 615 patients with LUAD for external validation. Finally, the single-cell dataset GSE131907 of LUAD was collected without additional processing and analyzed according to the original parameters (14). We used the “seurat” package to preprocess and analyze the single-cell data. In short, we log-normalized the data by “NormalizeData”. Subsequently, 1500 feature variables in the dataset were identified by “FindVariableFeatures”. PCA analysis was performed on the data set based on the 1500 feature variables. Finally, neighbors and cell clusters were identified by the first 40 principal components.

### Cell culture and transfection

We obtained two lung cancer cell lines (HCC827 and A849) and a normal lung epithelial cell line (16HBE) from GAINING-Bioscience (China). These cells were cultured in DMEM (Biological, Israel) supplemented with 10% heat-inactivated FBS, 1% penicillin, and 1% streptomycin. The cells were grown at a constant temperature of 37°C and with 5% CO2. We used Lipofectamine 8000 reagent (Invitrogen, USA) for transient transfection of siRNA according to the manufacturer’s instructions, aiming to suppress the expression of specific genes. The siRNAs used and their corresponding blank controls were as follows: si-FADD: GGAAGAAGACCTGTGTGCAGCATTT; siNC: GGAAGAAGTCCGTGTCGACAGATTT.

### qRT−PCR

Whole RNA was extracted using TRIzol reagent (Invitrogen, CA, USA) according to the instructions. cDNA of FADD was amplified according to the following primers: Forward: CATCTACCTCCGAAGCGTCC; Reverse: GGGCTACCTTCCTGGAGAGA. The 7500 Fast Real-time PCR system (Thermo Fisher, USA) was used for gene quantification according to the instructions, and the internal reference was GAPDH.

### Cell counting Kit-8 assay

Following the operating manual of the kit, we applied the Cell Counting Kit-8 (CCK-8, Bioss, China) to detect the growth activity of various LUAD cell lines. An enzyme-linked immuno-absorbance assay (BioTek, USA) was used to reflect the cell numbers by measuring the absorbance at 450 nm during cell growth.

### FADD-related functional enrichment

To assess the significant biological pathways of subgroups with different levels of FADD, we first identified significantly differentially expressed genes (DEGs) among FADD subgroups based on a threshold of fold change>2 and adjusted p value<0.05 utilizing the “limma” package. Functional annotation and enrichment analysis of DEGs were achieved through the Metascape online database (https://metascape.or). The differentially enriched pathways among different FADD subgroups were subsequently analyzed and identified by GSEA software (version 4.0.1) based on the KEGG background database.

### Dissecting the immune microenvironment associated with FADD

We systematically analyzed the heterogeneity of the immune microenvironment in different FADD subgroups from different perspectives. First, we evaluated the differences in cell types among different FADD subgroups from a single-cell perspective and assessed the cellular interaction pathways between cells with different FADD levels using the “CellChat” R package (15). The immune scores between different FADD subgroups were generated by the “ESTIMATE” algorithm (16). Subsequently, ssGSEA was executed by the “gsva” package to assess the relative activity of tumor immune-related pathways among different FADD subgroups (17). Finally, the abundance of immune cell infiltration among FADD subgroups was assessed by the “Cibersort” algorithm containing the reference expression profiles of 22 immune cells (18).

### Dissecting FADD-associated gene mutations

We collected homologous recombination defect (HRD) scores and MSI scores of different LUAD patients in the TCGA cohort from previous studies and assessed the differences between different FADD subgroups by “ggpubr” (19). We processed the raw maf files using “maftools” and calculated the tumor mutation burden (TMB) of individual patients after excluding nonsense mutated fragments (20). We classified significantly mutated genes according to a threshold of mutation number >45 and evaluated their differences and mutational cooccurrence among different FADD subgroups.

### Assessment of treatment sensitivity among FADD subgroups

Based on the ridge regression algorithm in the R package “pRRhetic”, we evaluated the differences in sensitivity of five first-line lung adenocarcinoma chemotherapeutic agents among different FADD subgroups (21). The results were generated as the patient’s half-maximal inhibitory concentration (IC50) values for a given drug, with lower IC50 values being more sensitive to treatment. We then assessed the sensitivity of patients to immunotherapy based on the level of immune cell exhaustion using the TIDE algorithm (22). Finally, we assessed the similarity of the expression profiles of the TCGA and Meta-GEO cohorts to the immunotherapy cohort by the subclass mapping algorithm and generated the sensitivity to anti-PD-1 and anti-CTLA-4 treatments.

### Statistical analysis

We used R software (Version 4.1.0) and GraphPad Prism 9.0 to analyze the data and draw figures. Appropriate t tests or Wilcoxon tests were applied to compare differences between the two subgroups according to the data structure. χ2 tests were used to compare differences in percentages. The significance of survival prediction was assessed by the log-rank test and univariate and multivariate Cox regression. The correlation of continuous variables was examined by Pearson’s coefficient. The threshold of significance was set at P<0.05.

## Results

### The pancancer perspective of FADD

**Figure 1:**
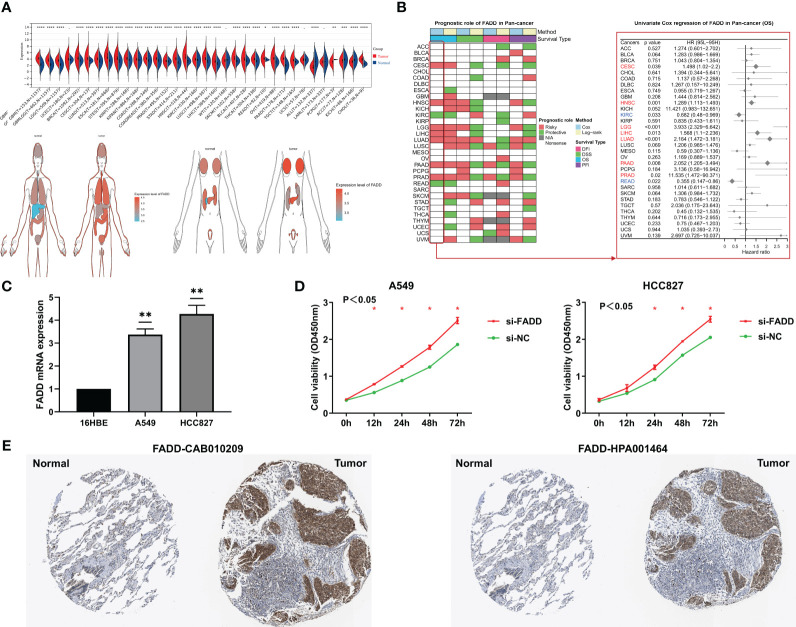
Analysis of FADD from a pan-cancer perspective **(A)** Differential expression of FADD in normal and tumor organs or tissues. **(B)** Log-rank and univariate Cox regression analysis of FADD in 32 solid tumors for survival. **(C)** FADD mRNA expression in LUAD cell lines (A549, and HCC827) compared with the normal lung cell line 16HBE by qRT−PCR. **(D)** The proliferation levels of lung cancer cell lines (A549, and HCC827) with knockdown of FADD by CCK-8. **(E)** Immunohistochemical staining analysis of FADD in normal lung and LUAD tissues from HPA database, left: CAB010209 antibody; right: HPA001464 antibody. *: P<0.05, **: P<0.01, ***: P<0.001, ****: P<0.0001, -: not significant.

In the TCGA database, the mRNA levels of FADD were significantly overexpressed in 27 solid malignancy tissues, including LUAD (P < 0.05, **Figure 1A**). Survival analysis from a pancancer perspective showed that FADD was a significant risk indicator for seven malignancies, including LUAD (**Figure 1B**). We further confirmed by qRT−PCR that the mRNA levels of FADD were significantly higher in two LUAD cell lines (A549 and HCC827) than in the normal lung tissue cell line 16HBE (**Figure 1C**). By CCK8 assay, we confirmed that FADD played a role in promoting tumor cell proliferation in LUAD cell lines (**Figure 1D**). At the protein level, we found significantly higher FADD levels in LUAD tumor tissues than in normal alveolar epithelium by two FADD antibody staining results (CAB010209 and HPA001464) in the HPA database (https://www.proteinatlas.org/) (**Figure 1E**).

### Prognostic potential of FADD in LUAD

**Figure 2:**
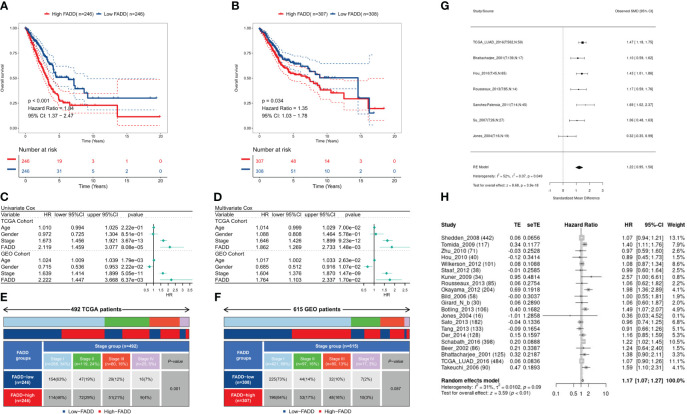
Prognostic potential of FADD in LUAD **(A)** KM survival curves for the high-FADD and low-FADD groups in the TCGA cohort. **(B)** KM survival curves for the high-FADD and low-FADD groups in the Meta-GEO cohort. **(C)** Univariate Cox regression analysis of FADD and OS in TCGA and meta-GEO cohorts. **(D)** Multivariate Cox regression analysis of FADD and OS in TCGA and meta-GEO cohorts. **(E)** Correlation analysis of FADD expression with stage in TCGA cohort. **(F)** Correlation analysis of FADD expression with stage in Meta-GEO cohort. **(G)** Meta-analysis of differential expression of FADD in LUAD normal and tumor tissues. **(H)** Meta-analysis of FADD in LUAD for predicting overall survival.

Survival analysis showed significantly worse survival in both TCGA-LUAD and GEO-LUAD datasets in patients with high FADD than in patients with low FADD (P<0.001, P=0.034, respectively; **Figures 2A, B**). Both univariate and multifactorial Cox regression analyses corrected for clinical characteristics showed that FADD could be an independent risk factor for OS in patients with LUAD (P<0.05, **Figures 2C, D**). Furthermore, by analyzing clinical characteristics, we found a significant positive correlation between FADD and stage in the TCGA dataset (P<0.001, **Figure 2E**) and a positive trend in the GEO dataset (P=0.087, **Figure 2F**). Comprehensive meta-analysis in the small cell lung cancer database (https://lce.biohpc.swmed.edu/lungcancer) showed that FADD was significantly elevated in tumor tissues (**Figure 2G**) and was an unfavorable prognostic indicator for OS in lung adenocarcinoma patients (**Figure 2H**). In addition, subgroup survival analysis showed excellent prognostic efficacy of FADD in patients with early-stage LUAD in the TCGA cohort, especially in patients with stage II LUAD and age >= 70 years (**Figure S1A**), whereas FADD in the GEO cohort showed good prognostic performance in patients younger than 70 years and in male patients (**Figure S1B**).

### Dissecting the functional differences at different FADD levels

**Figure 3:**
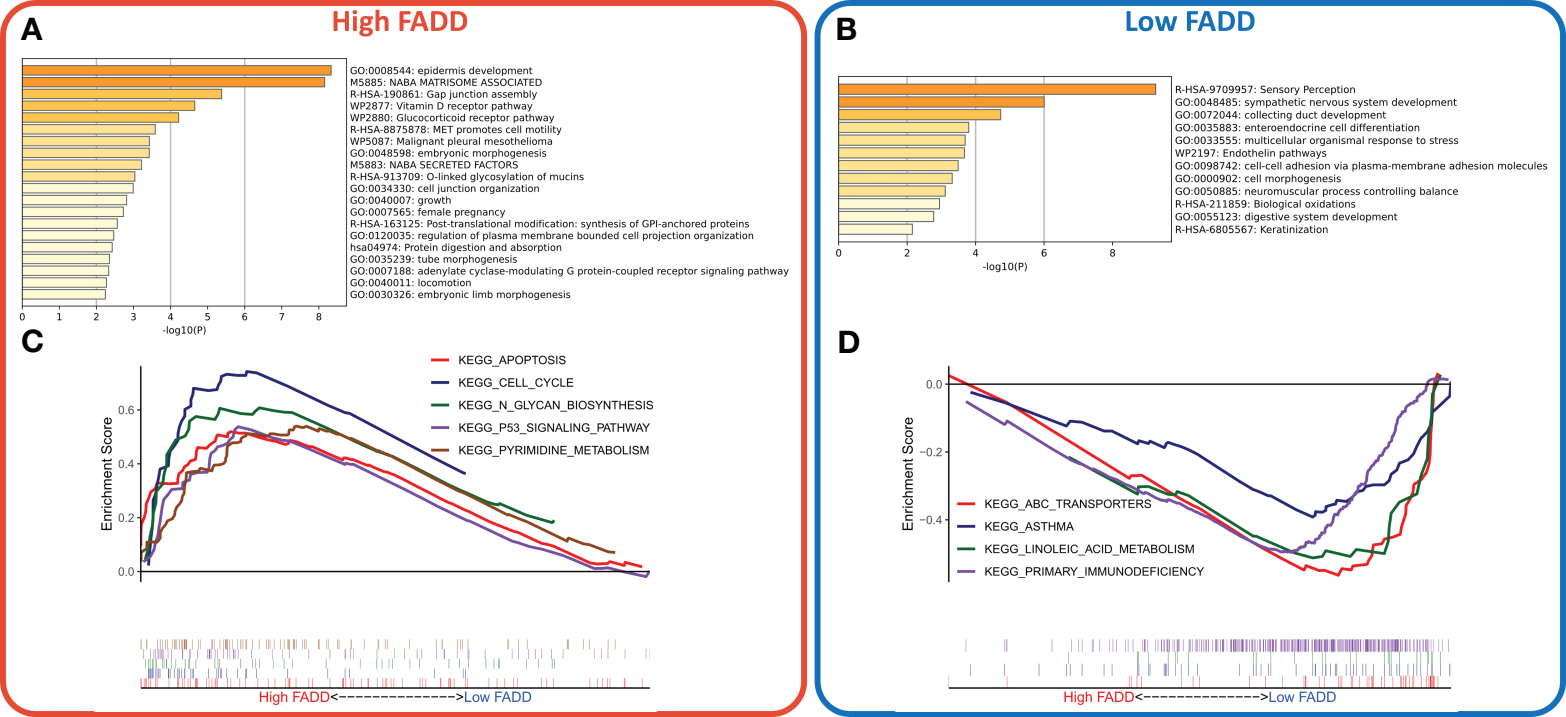
Construction and evaluation of a CDSig-based nomogram **(A)** Bar plot showed the biological pathways of upregulated gene enrichment in the high FADD group. **(B)** The barplot showed the biological pathways of upregulated gene enrichment in the low FADD group. **(C)** GSEA analysis revealed the top five enriched KEGG pathways in the high FADD group. **(D)** GSEA analysis revealed the top five enriched KEGG pathways in the low FADD group.

We identified a total of 201 DEGs, of which 91 DEGs were upregulated in the high FADD subtype and 110 DEGs were upregulated in the low FADD subtype. Functional enrichment analysis showed that the upregulated genes in the high FADD subtype mainly modulated cell growth, cell junctions, and epidermal development (**Figure 3A**), while the upregulated genes in the low FADD subtype mainly modulated biological oxidation and cellular morphogenesis (**Figure 3B**). GSEA revealed that the pathways enriched in the high FADD subtype were cell cycle, pyrimidine metabolism, P53 signaling pathway, and apoptosis (**Figure 3C**). In contrast, the pathways enriched in the low FADD subtype were primary immune defense, ABC transport, and asthma (**Figure 3D**). In conclusion, these results suggest that patients with low FADD subtypes have a stronger oxidative stress response and immune activity, whereas tumor cells with high FADD subtypes have a dysregulated cell cycle with hyperactive cell division and cell proliferation.

### Single-cell heterogeneity at different FADD levels

**Figure 4:**
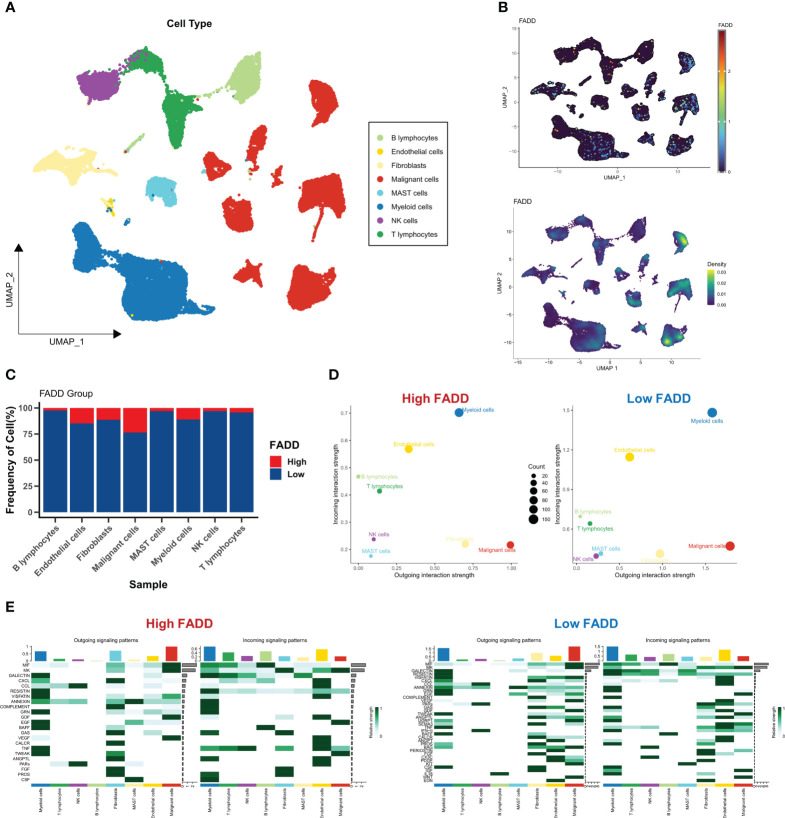
Single-cell perspective analysis of CDSig **(A)** Umap demonstrates 8 cell subgroups in LUAD patients. **(B)** The expression levels of CDSig in different cells. **(C)** The proportion of cells in different FADD subgroups. **(D)** Overall cellular communication pairs in cells with high FADD (left) and low FADD (right). **(E)** Specific communication pathways between different cells with high FADD (left) and low FADD (right).

We first assessed the microenvironmental heterogeneity of different levels of FADD from a single-cell perspective. We identified eight cell subtypes according to the original parameters (**Figure 4A**). We then found that FADD was highly expressed in most malignant and myeloid cells (**Figure 4B**). Specifically, low FADD cells were more predominant in B cells, NK cells, and T cells, while high FADD cells were more predominant in malignant cells and endothelial cells (**Figure 4C**). We identified significant cellular communication pairs based on a threshold of P<0.05, and the results showed that cells with low FADD expression had more cellular communication activity overall, especially myeloid and malignant cells (**Figure 4D**). **Figure 4E** shows the detailed communication pathways between different FADD cell subgroups, with fewer active pathways in high FADD cells and more active pathways in low FADD cells. Most of the communication pathways are associated with antitumor immunity (e.g., CXCL, CCL, TNF, etc.) (**Figure 4E**). In addition, malignant cells significantly received VEGF signaling (**Figure 4E**).

### Dissecting immune infiltration heterogeneity at different FADD levels

**Figure 5:**
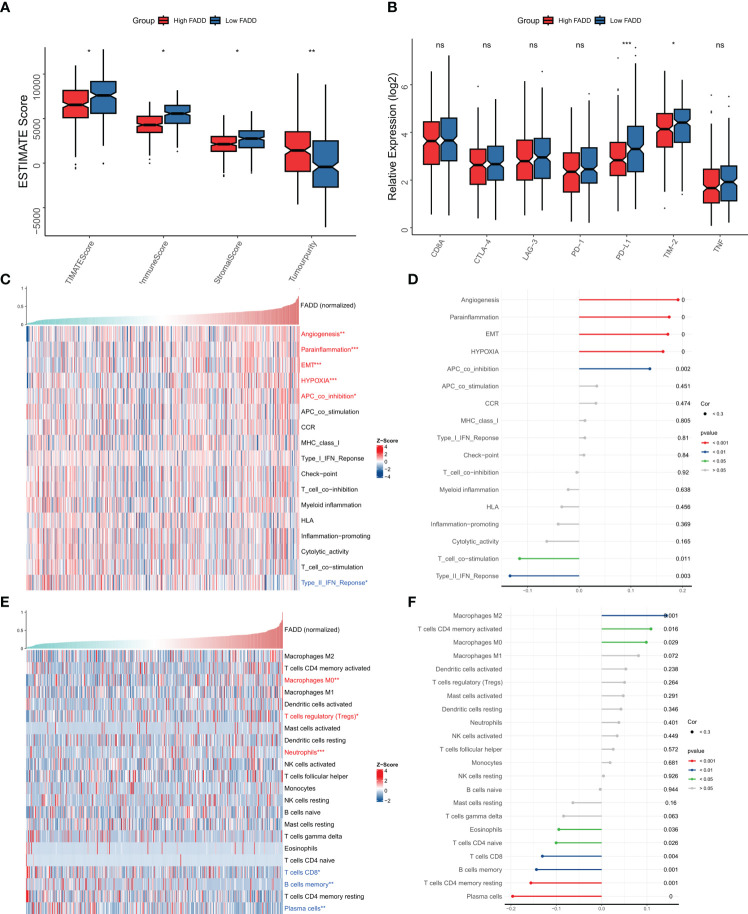
Immune microenvironment of different FADD subgroups **(A)** Box plots showing ESTIMATE results between different FADD subgroups. **(B)** Box plot showing immune checkpoint expression between different FADD subgroups **(C)** The landscape of immune-related pathways in different FADD subgroups. **(D)** Correlation between the immune-related pathways and expression level of FADD. **(E)** The landscape of immune cell infiltration in different FADD subgroups. **(F)** Correlation between the immune cell infiltration and expression level of FADD. *: P<0.05, **: P<0.01, ***: P<0.001; ns, not significant.

We then assessed the heterogeneity of immune infiltration due to different FADD levels from a Bulk-seq perspective. As assessed by the ESTIMATE algorithm, low FADD subtypes had higher immune scores, while high FADD subtypes had higher tumor purity (**Figure 5A**). We then examined the expression differences of seven classical immune checkpoints and therapeutic targets (CD8A, CTLA-4, LAG-3, PD-1, PD-L1, TIM-2, TNF) and found that TIM-2 and PD-L1 were significantly upregulated in the low FADD subtype (**Figure 5B**). Subsequently, by the ssGSEA algorithm, we found that the type II interferon response pathway was upregulated in the low FADD subgroup. While angiogenesis, EMT, hypoxia, paracrine immunity, and APC-coinhibitory pathways were upregulated in the high FADD subgroup (**Figure 5C**), the corresponding correlation analysis is shown in **Figure 5D**. Finally, Cibersort results showed that CD8 T cells, plasma cells, and memory B cells were upregulated in the low FADD subtype, whereas Tregs and M0 macrophages were upregulated in the high FADD subtype (**Figure 5E**), and the corresponding correlation analysis is shown in **Figure 5F**. Notably, M2 macrophages and FADD expression were significantly positively correlated (**Figure 5F**). In conclusion, these results suggest that low FADD levels may indicate “hot” tumors with active antitumor immunity. In contrast, high FADD levels were positively correlated with Tregs and M2 macrophages, which may represent immunosuppressed “cold” tumors.

### Assessment of FADD-associated genetic mutations

**Figure 6:**
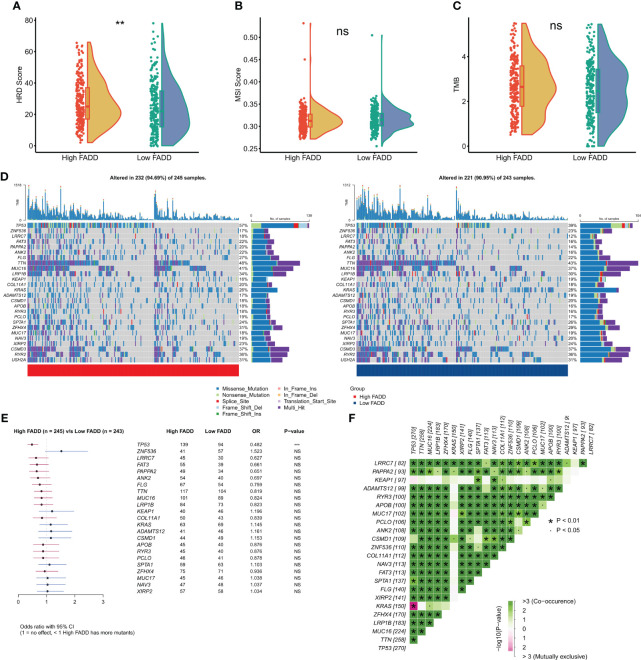
Genomic mutations in different FADD subgroups **(A)** Violin plot displayed the difference in **(A)** HRD score, **(B)** MSI score, and **(C)** TMB between different FADD subgroups. **(D)** Oncoplot showed the mutation landscape of high-frequency mutant genes between different cells with high FADD (left) and low FADD (right). **(E)** Forestplot showed statistically significant differences in high-frequency mutated genes between the high- and low-FADD subgroups. **(F)** Analysis of co-occurrence between high-frequency mutated genes **: P<0.01, ***: P<0.001; ns, not significant.

We first assessed overall the differences in two indicators of genomic mutations at different FADD levels. The results showed that patients with high FADD had significantly higher HRD scores (**Figure 6A**), but there was no significant difference in MSI scores between the two FADD subtypes (**Figure 6B**). We calculated the TMB for each patient and showed that the high FADD subtype had a higher TMB, but the difference in TMB between the two subtypes was not significant (**Figure 6C**). We processed the raw mutation data with the maftools package, and the oncoplot showed the difference in mutation profiles between the two FADD subtypes for a total of 26 high-frequency mutated genes (**Figure 6D**). The chi-square test showed an increased frequency of TP53 mutations in the high FADD subtype but no significant differences in the other 25 high-frequency mutated genes (**Figure 6E**). The co-occurrence analysis showed that all high-frequency mutated genes were highly co-occurring and showed significant concordance (**Figure 6F**).

### FADD can guide clinical decision-making in patients with LUAD

**Figure 7:**
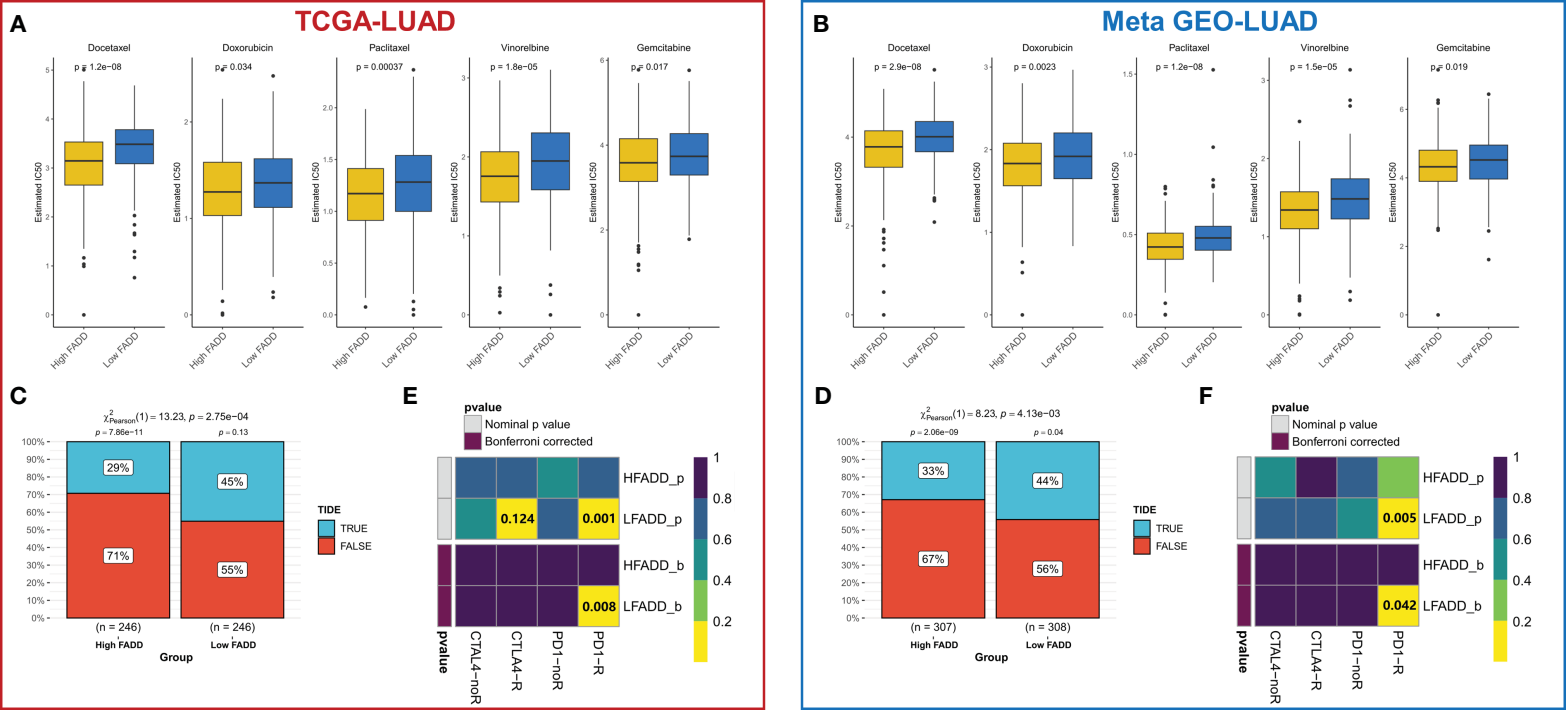
Treatment sensitivity among different FADD subgroups **(A)** Box plots displayed the predicted IC50 values for five first-line drugs of LUAD in high- and low-FADD groups in the TCGA cohort. **(B)** Box plots displayed the predicted IC50 values for five first-line drugs of LUAD in high- and low-FADD groups in the Meta-GEO cohort. **(C)** Response rates to immunotherapy in different FADD subgroups based on TIDE predictions in the TCGA cohort. **(D)** Response rates to immunotherapy in different FADD subgroups based on TIDE predictions in the Meta-GEO cohort. **(E)** Predicting the sensitivity of patients in high and low FADD groups to PD1 and CTLA4 treatment regimens by subclass mapping in the TCGA cohort. **(F)** Predicting the sensitivity of patients in high and low FADD groups to PD1 and CTLA4 treatment regimens by subclass mapping in the Meta-GEO cohort.

We then evaluated the efficacy of FADD in predicting sensitivity to chemotherapy and immunotherapy. Using ridge regression calculations, we found that patients with high FADD expression had lower IC50 values for the five first-line agents for LUAD (cisplatin, docetaxel, gemcitabine, paclitaxel, and vincristine), indicating that patients with high FADD are more sensitive to conventional chemotherapy (**Figure 7A**). The same results were observed in the externally validated Meta-GEO cohort (**Figure 7B**). The heterogeneity of the immune microenvironment due to different FADD levels suggested the existence of differences in immunotherapy sensitivity; therefore, we first evaluated the response rate of patients with different FADD levels to immunotherapy using the TIDE algorithm. The results showed that patients in the low FADD group in the TCGA-LUAD cohort had a higher response rate to immunotherapy (P=<0.001, **Figure 7C**). In the GEO-LUAD cohort, patients in the low FADD group also responded more to immunotherapy (P<0.001, **Figure 7D**). Finally, after subclass mapping assessment of the transcriptome, patients in the low FADD group in the TCGA and GEO cohorts were predicted to be more sensitive to anti-PD1 therapy (TCGA: FDR=0.008; GEO: FDR=0.042) (**Figures 7E, F**).

## Discussion

Lung cancer remains a major healthcare challenge worldwide today, and LUAD is the most common histopathological subtype of lung cancer (1). Although advances in medical technology and clinical protocols have driven advances in diagnostic and therapeutic approaches, the morbidity and mortality rates of LUAD remain high (23). FADD is a widely studied regulator of apoptosis, and recent novel findings propose a close relationship between FADD and cancer immunity (24). FADD not only enhances the activity of effector immune cells (especially T cells) (10, 11) but also reduces immune escape (12). Therefore, FADD has emerged as a promising new target for tumor immunotherapy. Our study defined two heterogeneous subtypes in LUAD based on FADD expression, one of which has more FADD expression and is defined as a high FADD subtype. The other subtype with low FADD expression was defined as the low FADD subtype. The subtype with high FADD expression has a dysregulated cell cycle that may lead to active tumor cell replication and proliferation. The subtype with low FADD expression is associated with stronger antitumor immunity. In addition, both subtypes showed heterogeneity in genomic mutation sites, clinical outcomes and immunotherapeutic response, revealing the potential of FADD as a novel target for precision and targeted medicine in LUAD.

These two subtypes exhibit different clinical features, and we observed that patients with a low FADD phenotype had significantly better overall survival than those with a high FADD phenotype and that more patients with advanced LUAD had the high FADD phenotype. Functional enrichment suggests that the high FADD phenotype is enriched in purine pyrimidine metabolism-related pathways and cell cycle-related pathways, which are hallmarks and one of the fundamental mechanisms of cancer, suggesting that high FADD is a dysregulated, hyperproliferative LUAD subtype (25, 26). Further analysis revealed a significant increase in angiogenic, EMT, and hypoxic pathway activity in patients with high FADD, which represents a positive correlation between high FADD and the malignant features of the tumor. Moreover, high FADD could indicate patients with higher malignancy of LUAD (27–29). In addition to the enrichment of immune-related pathways in patients with low FADD expression, immune infiltration analysis also suggested a higher infiltration of effector immune cells (CD8+ T cells) and B cells in the low FADD phenotype (30). As important components of the antitumor immune system, the enrichment of CD8+ T cells and B cells provides an additional source of power for patients with low FADD to exert stronger antitumor immunity and may ultimately lead to a better prognosis for patients with low FADD (31, 32). Notably, higher FADD expression is positively associated with increased infiltration of suppressive immune cells (Tregs and M2 macrophages), which may lead to suppressed antitumor activity and immune escape of active tumor cells in patients with high FADD (33, 34). The high FADD subtype exhibits features of immune exhaustion and ultimately leads to a poor prognosis.

One of the features of tumorigenesis is the abnormal accumulation of genetic mutations, and therefore, mutational differences among different FADD subtypes may contribute to the eventual phenotypic differences (35). We found that high FADD was associated with a significantly higher HRD score, and existing reports suggest that an increased HRD score can identify immunophenotypically “cold” tumors, which is consistent with our findings (36). In addition, higher HRD scores were also associated with increased sensitivity to platinum-based chemotherapy (37). Although we failed to find significant differences in TMB among patients with different FADD levels, we found that patients with high FADD had more TP53 mutations. TP53 is a reported classical cancer suppressor gene that is highly mutated in most patients with LUAD (38). Therefore, we infer that more cumulative TP53 mutations are also a factor contributing to the worse prognosis of patients with high FADD.

Taken together, our results suggest that FADD represents different states of the “cold” and “hot” tumor microenvironment and may contribute to the heterogeneity of tumor treatment sensitivity. Accordingly, our subsequent analysis focused on the differences in sensitivity to conventional chemotherapy and immunotherapy among patients with different FADD levels. We first found that patients with high FADD had a lower IC50 for first-line chemotherapeutic agents for LUAD, and therefore, patients with high FADD were more amenable to conventional chemotherapy. Our GSEA suggests that tumor cell cycle pathways are enriched and cell proliferation is active in high FADD, and the active cell cycle provides more targets for conventional chemotherapeutic agents, thus leading to greater sensitivity to chemotherapy (39). After evaluation by the TIDE algorithm and the subclass mapping algorithm, we were able to determine that patients with low FADD have a higher response rate to anti-PD-1 inhibitors. Notably, our results suggest that low FADD is associated with greater PD-1 and PD-L1 expression and that patients with low FADD have immune-active tumor microenvironments. Therefore, our prediction for the applicability of immunotherapy in patients with low FADD is reliable. In conclusion, FADD can be applied to specify individualized treatment regimens for LUAD patients in the clinic, with high FADD more applicable to conventional chemotherapy and low FADD more applicable to novel immunotherapy represented by anti-PD-1 inhibitors.

## Conclusion

In conclusion, this study suggests that upregulated FADD is associated with worse prognosis, immune exhaustion, and tumor malignancy in patients with LUAD. Furthermore, FADD could be a useful indicator for assessing sensitivity to chemotherapy and immunotherapy. Therefore, FADD has the potential to become a new target for precision medicine and targeted therapy for LUAD.

## Data availability statement

The original contributions presented in the study are included in the article/**Supplementary Material**. Further inquiries can be directed to the corresponding authors.

## Author contributions

LW and FL designed and supported this study, MH analyzed the data and drafted the manuscript, YH and JX drew figures and improved the manuscript, YZ and XC collected the data and completed the experiment. All authors contributed to the article and approved the submitted version.

## Supplementary material

Supplementary Figure 1 Subgroup survival analysis of FADD Subgroup KM survival curves of FADD in TCGA **(A)** and Meta-GEO **(B)** cohorts.
