# Supplementary figures and images for: Upregulated FADD is associated with poor prognosis, immune exhaustion, tumor malignancy, and immunotherapy resistance in patients with lung adenocarcinoma
Source: Front Oncol. 2023 Aug 21;13:1228889. doi: 10.3389/fonc.2023.1228889 (PMC10476093; doi:10.3389/fonc.2023.1228889)

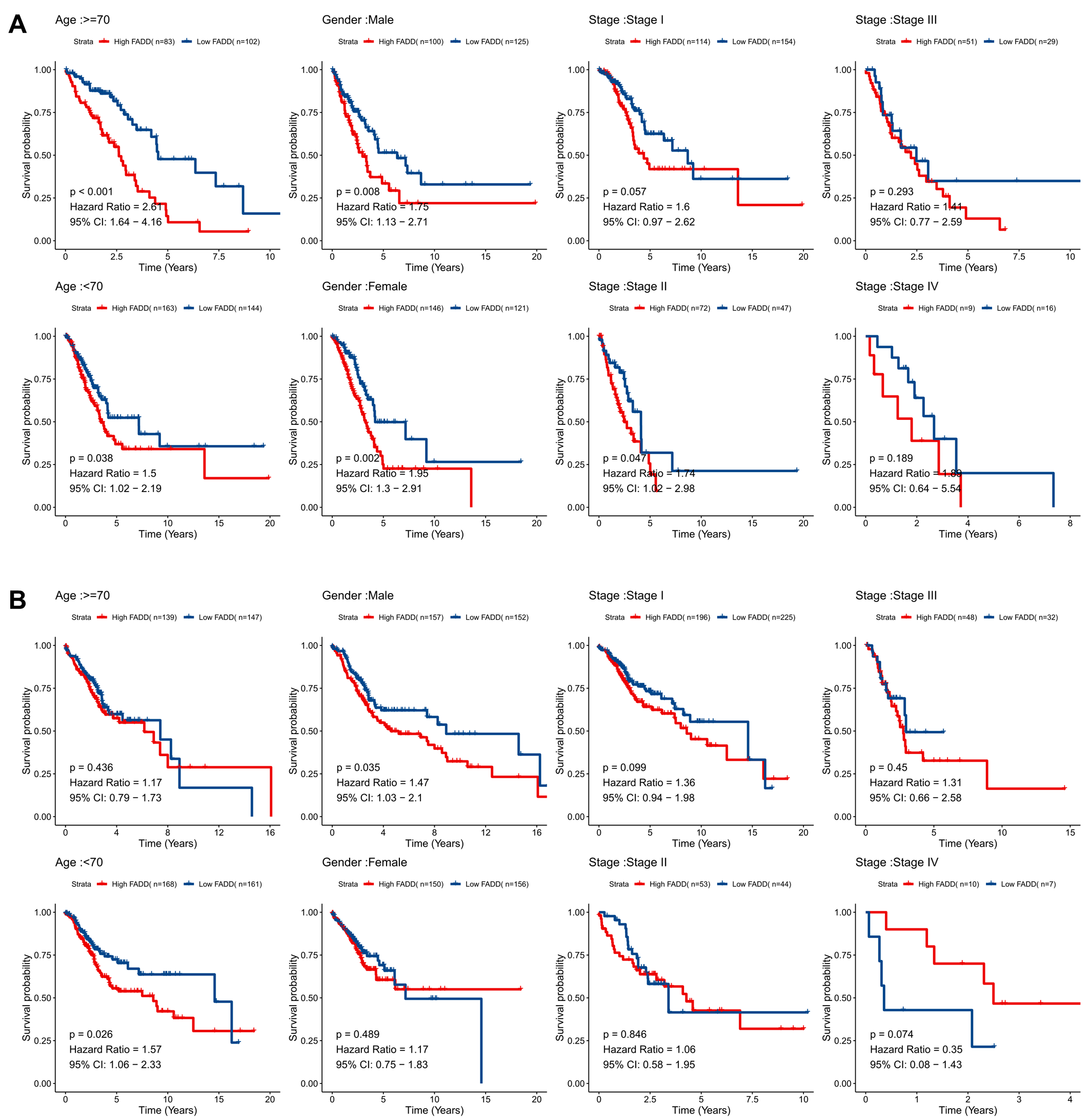

Supplement: Supplementary Figure 1 — Subgroup survival analysis of FADD Subgroup KM survival curves of FADD in TCGA (A) and Meta-GEO (B) cohorts. [file Image_1.tif]
